# *Saccharomyces cerevisiae boulardii* accelerates intestinal microbiota maturation and is correlated with increased secretory IgA production in neonatal dairy calves

**DOI:** 10.3389/fmicb.2023.1129250

**Published:** 2023-09-19

**Authors:** Lautaro Rostoll Cangiano, Clothilde Villot, Rocio Amorin-Hegedus, Nilusha Malmuthuge, Robert Gruninger, Le Luo Guan, Michael Steele

**Affiliations:** ^1^Department of Animal Biosciences, University of Guelph, Guelph, ON, Canada; ^2^Lallemand Animal Nutrition, Blagnac, France; ^3^Lallemand Animal Nutrition, Milwaukee, WI, United States; ^4^Genetics Institute, University of Florida, Gainesville, FL, United States; ^5^Lethbridge Research and Development Centre, Agriculture and Agri-Food Canada, Lethbridge, AB, Canada; ^6^Department of Agricultural, Food and Nutritional Science, University of Alberta, Edmonton, AB, Canada

**Keywords:** intestinal immunity, microbiome, microbial colonization, calf health, immune development

## Abstract

Neonatal calves have a limited capacity to initiate immune responses due to a relatively immature adaptive immune system, which renders them susceptible to many on-farm diseases. At birth, the mucosal surfaces of the intestine are rapidly colonized by microbes in a process that promotes mucosal immunity and primes the development of the adaptive immune system. In a companion study, our group demonstrated that supplementation of a live yeast probiotic, *Saccharomyces cerevisiae boulardii* (SCB) CNCM I-1079, to calves from birth to 1 week of age stimulates secretory IgA (sIgA) production in the intestine. The objective of the study was to evaluate how SCB supplementation impacts the intestinal microbiota of one-week-old male calves, and how changes in the bacterial community in the intestine relate to the increase in secretory IgA. A total of 20 calves were randomly allocated to one of two treatments at birth: Control (CON, *n* = 10) fed at 5 g/d of carrier with no live yeast; and SCB (*n* = 10) fed at 5 g of live SCB per day (10 × 109 CFU/d). Our study revealed that supplementing calves with SCB from birth to 1 week of age had its most marked effects in the ileum, increasing species richness and phylogenetic diversity in addition to expediting the transition to a more interconnected bacterial community. Furthermore, LEfSe analysis revealed that there were several differentially abundant taxa between treatments and that SCB increased the relative abundance the family *Eubacteriaceae*, *Corynebacteriaceae*, *Eggerthellaceae*, *Bacillaceae*, and *Ruminococcaceae*. Furthermore, network analysis suggests that SCB promoted a more stable bacterial community and appears to reduce colonization with *Shigella*. Lastly, we observed that the probiotic-driven increase in microbial diversity was highly correlated with the enhanced secretory IgA capacity of the ileum, suggesting that the calf’s gut mucosal immune system relies on the development of a stable and highly diverse microbial community to provide the necessary cues to train and promote its proper function. In summary, this data shows that supplementation of SCB promoted establishment of a diverse and interconnected microbiota, prevented colonization of *Escherichia Shigella* and indicates a possible role in stimulating humoral mucosal immunity.

## 1. Introduction

At birth, neonatal calves have a limited capacity to initiate immune responses, and the immune system gradually matures during the first months of life, concurrent with microbial colonization and development (Wopereis et al., 2014). Although less studied in calves, microbial colonization of the gastrointestinal tract (GIT) during early life has been extensively reported in other mammalian species to be critical for normal immune and intestinal development (Hooper et al., 2012). The process of colonization is highly dynamic and modulated by several environmental and maternal factors that include colostrum and milk consumption, maternal and environmental microbes, diet, antimicrobial exposure, and weaning (Wopereis et al., 2014). During establishment of the intestinal microbiota there is a suppression of adaptive immunity to promote tolerance to the increasing load of bacteria that reaches the intestine, and the amount of antigen that is encountered by immune cells (Belkaid and Hand, 2014; Yang et al., 2020). Calves are highly dependent on successful passive transfer of immunoglobulins (Ig) from colostrum since there is no transfer of Ig through placenta and their adaptive immune system is not fully developed. Failure of passive transfer results in increased morbidity and mortality (Urie et al., 2018), with diarrhea accounting for over half of reported illnesses and one-third of deaths (Urie et al., 2018; Winder et al., 2018). Traditionally, antimicrobials are used to treat and prevent diarrhea. However, variable efficacy of antimicrobials, risk of developing antimicrobial resistance, and the long-term impacts on animal health and performance make this practice unsustainable (Windeyer et al., 2014; Aghakeshmiri et al., 2017; Buss et al., 2021). As a mean to minimize antimicrobial use, preventative approaches should be emphasized and alternatives to antimicrobials need to be pursued.

As previously mentioned, the gut microbiota modulates mucosal immunity and primes the development of the adaptive immune system during early life. In addition, the gut microbiome has greater plasticity in early life and perturbations to the intestinal microbial at this stage of life can have long-term health consequences (Laforest-Lapointe and Arrieta, 2017). The same is thought to occur in calves, as pre- (Marquez, 2014) and probiotics (Abe et al., 1995) exert the greatest effects when supplemented during the first weeks of life (Cangiano et al., 2020). This could be directly related to the instability of their microbial communities, whereas later in life the microbiome is stable and more difficult to influence (Malmuthuge et al., 2015). Hence, modulating the gut microbiome during early life, by supplementing microbial-based products, could become a sustainable option to enhance mucosal immunity and ultimately improve calf health (Malmuthuge and Guan, 2017). Previously, *Saccharomyces cerevisiae boulardii* (SCB) has been shown to reduce the incidence of severe diarrhea in pre-weaned calves (Villot et al., 2019) and increase average daily gain (ADG) after weaning (Renaud et al., 2019). In a companion study, it was shown that SCB supplementation to newborn calves from birth stimulates secretory IgA (sIgA) production in the intestine at 1 week of age (Villot et al., 2020), suggesting that this yeast probiotic can prime mucosal immunity. Secretory IgA is the most abundant immunoglobulin in mucosal secretions (Pabst et al., 2016) and represents one of the main non-inflammatory defense mechanisms of the gut along with antimicrobial peptides. It generates an adaptive immune response that prevents adhesion and invasion by microorganisms, neutralizing them and forcing its clearance out of the gastrointestinal tract (Mantis et al., 2011). The enhancement of humoral immunocompetence and increased secretion of IgA by SCB supplementation has also been demonstrated in germ-free mice models (Rodrigues et al., 2000; Martins et al., 2009), in normal BALB/C mice (Qamar et al., 2001), and broiler chickens (Rajput et al., 2013). However, the exact molecular mechanisms of action and the secreted molecules that confer its protective and immunomodulatory effects remain only partially understood. It has been shown that SCB reduces dendritic cell activation in cells culture, and promotes IL-10 secretion while reducing IL-6 and TNF-α, ultimately reducing T cell proliferation (Thomas et al., 2009). In addition, SCB reduces oxygen availability in the neonatal gut, accelerating the colonization of the intestine with strict anaerobic bacteria and stimulating microbial diversity. This in turn provides the necessary signals that stimulate intestinal maturation (Pais et al., 2020). Lastly, SCB administration after antimicrobial therapy helps restore the intestinal microbiota diversity faster in mice (Barc et al., 2008; Moré and Swidsinski, 2015). Therefore, we hypothesized that supplementation of SCB to newborn calves could accelerate intestinal microbial maturation, providing the necessary signals to stimulate the intestinal naïve B cells to become activated and increase their capacity to secrete IgA. The objective of the study was to evaluate how SCB supplementation impacts the intestinal microbiota of one-week-old male calves, and how changes in the bacterial community in the GIT relate to the increase in secretory IgA secretion observed our companion study.

## 2. Materials and methods

### 2.1. Animals, housing, treatments, and experimental design

The experiment was conducted as per the guidelines of the Canadian Council of Animal Care (CCAC, 1993) at the Dairy Research and Technology Center of the University of Alberta (Edmonton, Alberta, Canada), and the methodology was previously described in our companion study (Villot et al., 2020). The animal use protocol was approved by the University of Alberta Animal Care Committee (Animal Use Protocol # 2645). A total of 20 singlet and naturally delivered Holstein bull calves born from May to August 2018 with a birth body weight (BW) between 35 and 55 kg were enrolled in this study. Calves were removed from the maternity pen at birth to avoid contact with the dam and moved to disinfected indoor pens (150 × 125 cm) bedded with a consistent amount of fresh straw to facilitate nesting. Animals included in this study presented healthy at birth based on general appearance, rectal temperature, umbilical appearance, and nasal discharge; none were vaccinated or treated with therapeutic fluids during the trial. Calves received two meals of standardized colostrum replacer (HeadStart, Saskatoon Colostrum Co., Ltd., Saskatoon, SK, Canada). Briefly, calves received two feedings of colostrum at 2 h after birth fed at 7% of birth BW, and at 12 h after birth fed at 3% of birth BW to receive an average amount of 180, and 120 g of IgG, respectively (Villot et al., 2020). Colostrum was bottle fed, and the remainder of the meal volume was tube-fed if the calves did not consume all of the meal within 30 min. The concentration of IgG and IgA delivered to the calves was measured in a pooled colostrum batch composed of subsamples of all the colostrum fed to the calves, which confirmed that all calves received the same amount of IgG and IgA from colostrum (Villot et al., 2019). Milk replacer was bottle-fed, and it was composed of 260 g/kg of crude protein, 160 g/kg of crude fat, and 4.58 Mcal/kg of metabolizable energy on a dry matter basis (Grober Animal Nutrition, Cambridge, Ontario, Canada). The initial volume of individual meals offered to each calf was 7.5% of their birth BW during the 2 first days of the experiment and increased to 8.5% of BW from d 3 to d 7.

A generalized randomized block design was used to evaluate the impact of SCB supplementation on bacterial colonization and its impact on the development of humoral immunity of Holstein bull calves. Calves were randomly assigned to one of two treatments according to their date of birth and initial BW: Control (CON, *n* = 10) fed at 5 g/d of carrier with no live yeast; and *Saccharomyces cerevisiae boulardii* (SCB, *n* = 10) fed at 5 g of live SCB per day (10 × 10^9^ CFU) from birth to 1 week of life. Both the CON and SCB treatments were fed once daily starting with the first colostrum feeding and subsequently in the morning milk replacer feeding until 1 week of age when calves were euthanized.

### 2.2. Intestinal tissue and digesta sampling

At 1 week of life, 3 h after the morning meal, calves were euthanized to collect tissue and digesta samples from the gastrointestinal tract. Calves were euthanized with a pentobarbital sodium injection (Euthanyl, Vetoquinol, Lavaltrie, QC, Canada) at 0.125 mL/kg of BW administered through a jugular catheter. Once the calf reached a surgical plane of anesthesia, exsanguination was performed. The esophagus and rectum were first ligated to prevent loss of digesta and the entire gut digesta were then removed with caution to avoid transfer of digesta between the different segments. Segments of the proximal jejunum, ileum, and colon, along with their digesta were collected according to Villot et al. (2020). Briefly, the proximal jejunum sample was taken 100 cm distal to the pylorus sphincter, the ileum segment was defined as 30 cm proximal to the ileocecal junction, and the colon segment was defined as 30 cm distal to the ileocecal junction. Zip-ties were placed on each end of the segments so that a sample of digesta could be collected. Intestinal digesta were removed from the sample using tweezers and placed in a 50-mL Falcon tube. The tissue was then washed in sterile PBS (pH 7.4) until clean (~3–4 washes) and placed in a sterile bag. Both gut digesta and tissue samples were immediately snap-frozen in liquid nitrogen and then stored at −80°C until further processing.

### 2.3. Quantification of SCB, secretory IgA and SCFA in intestinal digesta

A primer set that targets the 26S rRNA gene of *S. cerevisiae* was used to estimate the copy numbers of SCB using real-time quantitative PCR (qPCR), according to Chang et al. (2007) and Villot et al. (2020). Briefly, the qPCR was performed in the high throughput Viia 7 Real-Time PCR System (Thermo Fisher Scientific, Waltham, MA) using SYBR green chemistry (Fast SYBR Green Master Mix, Applied Biosystems, Foster City, CA) with specific primers (Forward: 5′- AGGAGTGCGGTTCTTTG −3′, and Reverse: 5′- TACTTACCGAGGCAAGCTACA −3′). Genomic DNA of SCB was extracted from the commercial probiotic yeast used in the study (ProTernative Milk; CNCM I-1079 strain) to generate a standard curve to estimate the gene copy number of *S. cerevisiae* in calf digesta from proximal jejunum, ileum, and colon. All qPCR reactions were performed using the optimized conditions described previously by Villot et al. (2020), and the quantity of *S. cerevisiae* was calculated from the standard curve.

Data on sIgA concentration in intestinal digesta, as well as the analysis procedures, are presented in Villot et al. (2019). Briefly, sIgA was measured in intestinal digesta from jejunum, ileum, and colon on d 7. The concentration of sIgA was analyzed using a sandwich ELISA technique with a commercial kit (Bethyl Laboratories Inc., TX), as per the manufacturer’s recommendations and as described by Villot et al. (2020). All samples were tested in duplicate, and samples with an intra and inter-assay CV greater than 10% were repeated.

The concentrations of short-chain fatty acids (SCFAs; acetic, propionic, butyric, and caproic) were determined via gas chromatography, as described by Schlau et al. (2012). Briefly, digesta samples were thawed and centrifuged at 13,000 rpm for 5 min at 4°C and the supernatant mixed with 25% meta-phosphoric acid (4:1; vol/vol). The samples were then centrifuged again at 13,000 rpm for 5 min at 4°C, and the supernatant was mixed with internal standard solution (5:1; vol/vol) before incubation at −20°C overnight. After incubation, samples were centrifuged at 19,000 × *g* at 4°C for 5 min and the supernatant transferred to vials. Samples were analyzed by gas chromatography (Varian, Palo Alto, CA) using a flame ionization detector and a capillary column (CP-WAX 58 FFAP 25 m 0.53 mm, Varian CP7767, Varian Analytical Instruments, Walnut Creek, CA).

### 2.4. DNA extraction, library preparation, 16S amplicon sequencing

Extraction of microbial genomic DNA was performed similarly to that previously reported by Yu and Morrison (2004). Briefly, the digesta and tissue samples (~0.3 g ± 0.1 g) were washed twice with Tris-EDTA buffer. After the addition of cell-lysis buffer containing 4% SDS, samples were subjected to physical disruption at 5,000 rpm for 3 min using Biospec Mini Beads Beater 8 (BioSpec, Bartlesville, OK), followed by incubation at 70°C for 15 min and centrifugation for 5 min at 16,000 × *g* at 10°C. The bead beating, incubation, and centrifugation steps were repeated, and impurities were removed from the supernatant using 10 M ammonium acetate, followed by DNA precipitation using isopropanol. After precipitation, DNA was further purified using QIAmp fast DNA stool mini kit (Qiagen Inc., Germantown, MD). The quantity and quality of DNA were evaluated using the NanoDrop 1000 spectrophotometer, and DNA was stored at −20°C until further use.

For library preparation and sequencing, a fragment of the 16S rRNA gene spanning the V1–V3 hypervariable region was amplified by PCR using dual indexed {universal bacterial primers [9F (5′-GAGTTTGATCMTGGCTCAG-3′); 515R (5′- CCGCGGCKGCTGGCAC- 3′); Kroes et al., 1999]}. The amplicons with targeted size (~480 bp) were purified from 1% agarose gel using a QIAEX II gel extraction kit (Qiagen Inc.). The quality and quantity of purified PCR products were checked using a NanoDrop 1000 (NanoDrop Technologies, Wilmington, DE) to ensure that the concentration of DNA from all samples was higher than 25 ng/μL. Sequencing libraries were prepared using the Nextera XT Index (Illumina) and sequenced on an Illumina MiSeq platform (2 × 300 bp) at Genome Quebec (McGill University, Montreal, QC, Canada).

### 2.5. Bioinformatics and statistical analysis

The adapter sequences were trimmed from the raw fastq files, and the trimmed reads were demultiplexed according to the samples using the bcl2fastq2 conversion software version 2.20.0. (Illumina). For bioinformatic analysis, the sorted reads were imported and processed using the Quantitative Insight into Microbial Ecology (QIIME2) package version 2021.2 (Bolyen et al., 2019). First, low-quality reads (Phred score < 20) and short (<100 bp) reads were filtered out. This was followed by denoising and merging using the plugin DADA2 to generate an amplicon sequence variant (ASV) feature table. Sequences shorter than 400 bp as well as chimeric sequences and singleton ASVs were excluded from further analyses. Taxonomic classification was performed in QIIME2 using a scikit-learn naïve Bayes classifier trained for the V1-V3 region. Next, taxonomic assignment was performed using the trained classifier using the SILVA database (SILVA release 132) with 99% identity for representative bacterial sequences. Alpha and beta diversity were performed using QIIME2 with a sampling depth of 15,000 for the digesta samples, and 7,000 for the tissue samples. Alpha-diversity analysis was conducted with standard diversity metrics accessed via QIIME2, including Chao1, Shannon index, and Faith index. A nonparametric analysis of variance (Kruskal-Wallis test) was used to test differences in α-diversity among treatment groups and to calculate *p*-values within QIIME2. Beta-diversity was measured by calculating the weighted and unweighted UniFrac distances using QIIME2. Non-metric multidimensional scaling (NMDS) was applied on the resulting distance matrices, and analysis of similarity (ANOSIM) were used to calculate *p*-values and to test differences in β-diversity among treatment groups for significance. Furthermore, the linear discriminant analysis of effect size (LEfSe; Segata et al., 2011) was used to evaluate ASVs differences between treatments. The data were displayed using linear discriminant analysis score plots for significant ASVs after Kruskal–Wallis and Wilcoxon tests. A size-effect threshold of 3.5 on the logarithmic LDA score was used to identify discriminating taxa. Finally, the relative abundance of discriminant taxa from the LEfSe analysis were compared between groups using among the groups, and Mann–Whitney *U* test was used to identify taxa that were significantly different.

Microbiota network analysis was performed at the phylum and at the genus level and separately for each treatment group. Genera with less than 0.1% mean relative abundance and less than 25% prevalence among the different samples were excluded. The ASV relative abundance table obtained from QIIME was imported into R package Phyloseq and converted into an adjacency matrix through the use of R package SPIEC-EASI (Kurtz et al., 2015). Next, the Meinshausen-Bühlman neighborhood selection method was used to calculate the conditional dependence between the different ASV pairs. This approach was developed to perform network analysis for compositional and sparse data, such as relative abundance of ASV data, which prevents indirect and spurious associations from being generated (Kurtz et al., 2015). The obtained matrices were visualized as networks at both Phylum and Genus levels. The networks model the relationships between different ASVs, where nodes represent ASVs, and edges represent their co-occurrence across different samples. In order to evaluate ASV centrality in the networks, their hub scores were calculated and extracted from the networks using the SPIEC-EASI hub_score() function, and ranked. The 10 species with the highest hub scores were defined as the keystone species in each group.

Lastly, non-parametric Spearman rank correlation coefficient analysis was used to test the relationship between secretory IgA in ileum digesta (sIgA), short chain fatty acids profile (acetate, propionate, caproate, and total SCFAs), alpha diversity measures (species richness, evenness, and phylogenetic diversity), with the bacterial communities differentially abundant between treatments and *Saccharomyces cerevisiae* gene copy numbers present in ileum digesta. Physiological data were published previously by Villot et al. (2020) in a companion paper. The resulting correlation matrix was visualized in a heatmap format generated by the corrplot package of R [Corrplot: visualization of a correlation matrix; R package version 4.1.2. 2021].

## 3. Results

### 3.1. Alpha and beta diversity measures

**Figure 1:**
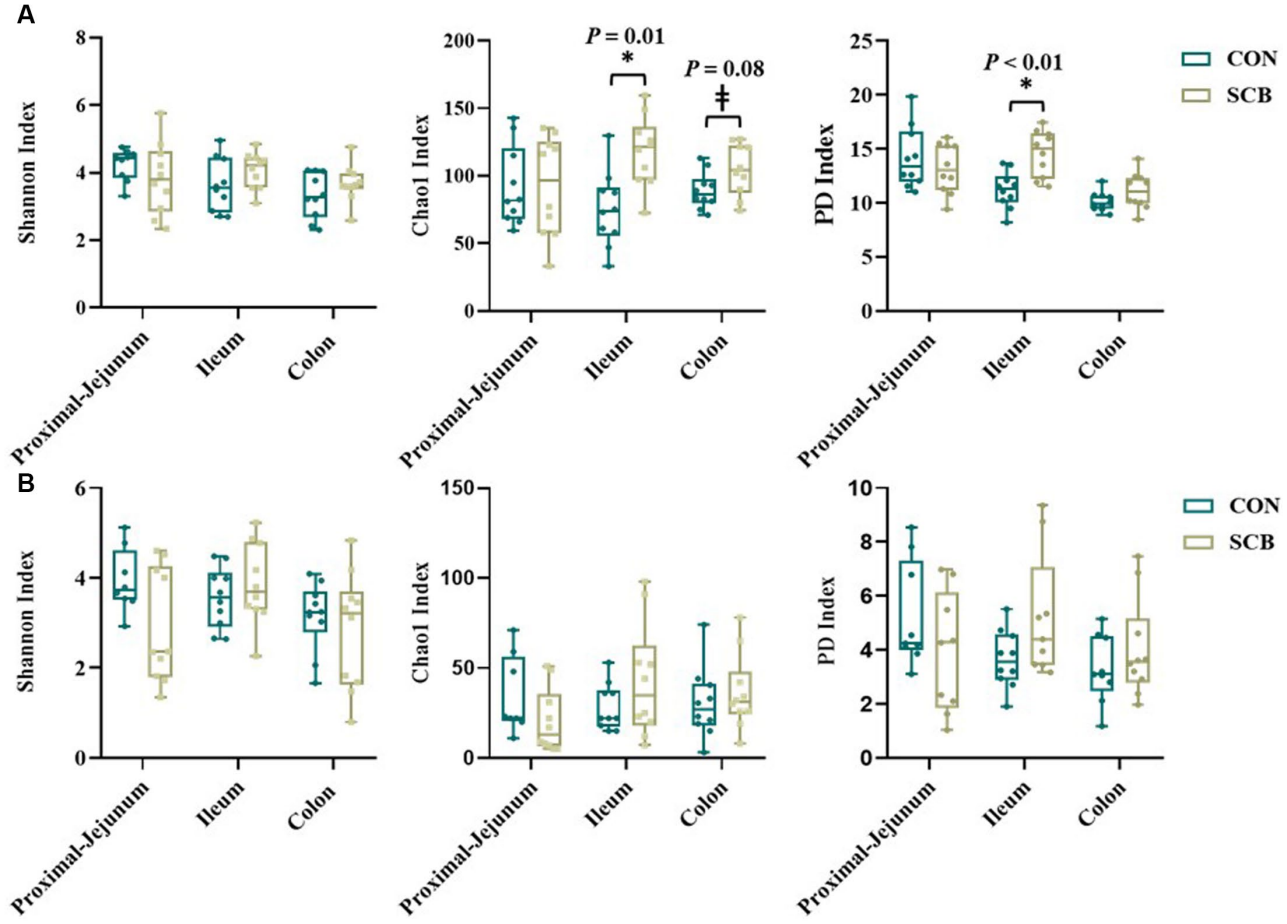
Alpha diversity index in digesta **(A)** and mucosa **(B)** at proximal jejunum, ileum, and colon of calves supplemented (SCB) or not (CON) from birth until 1 week of age with *Saccharomyces cerevisiae boulardii* CNCM I-1079 at 10 × 10^9^ cfu/d. Species richness and diversity index were measured with three different matrices: Chao1, Shannon index, and Faith phylogenetic diversity (PD index). * Indicates a significant difference between CON and SCB groups (*p* < 0.05), and ± denotes a tendency for a significant difference between CON and SCB groups (*p* < 0.10). The lines, boxes, and whiskers in the box plots represent the median, and 25th, and 75th percentiles, and the min-to-max distribution of replicate values, respectively.

**Figure 2:**
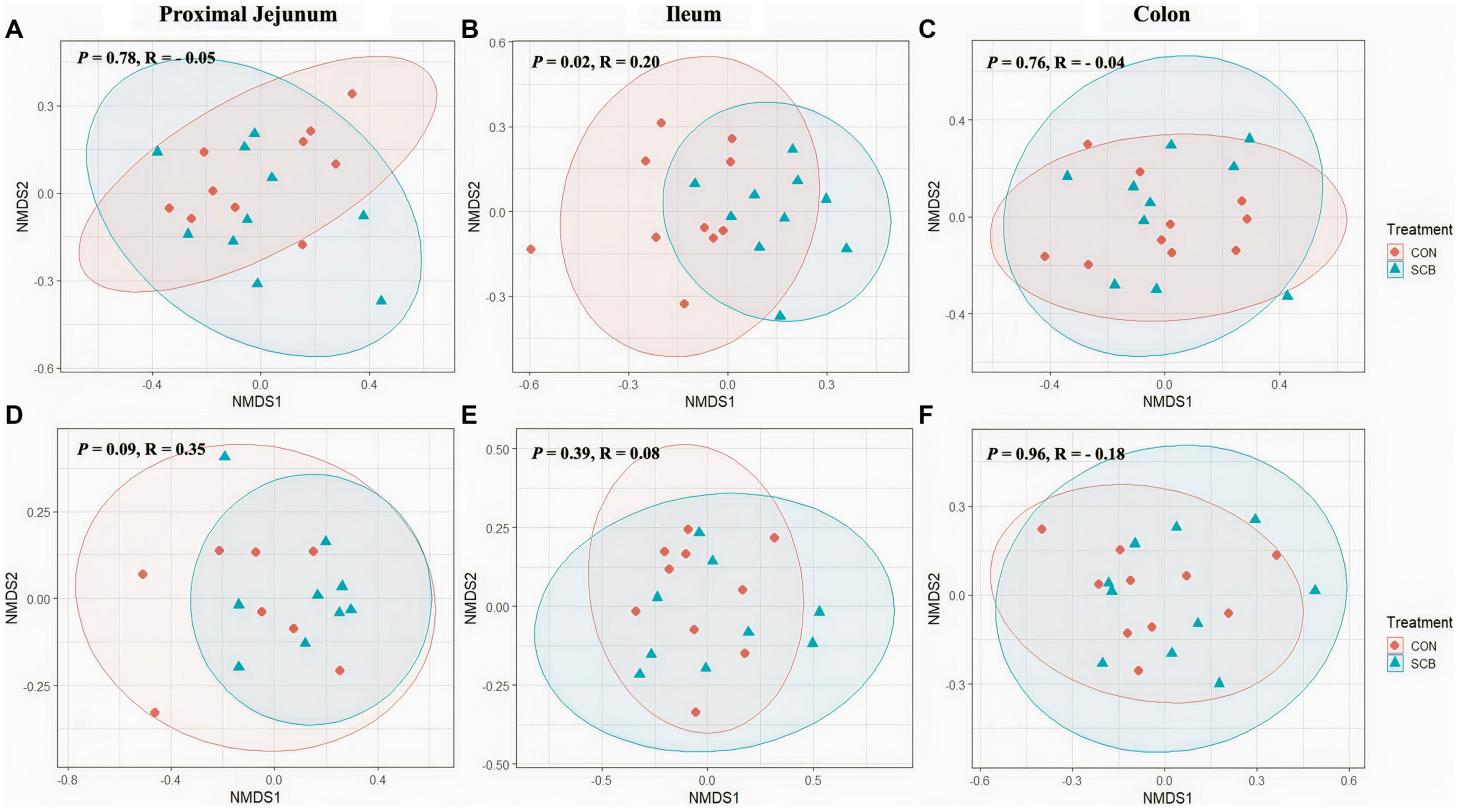
Non-Metric Multidimensional Scaling Plots (NMDS) based on unweighted unifrac distances in digesta **(A–C)** and mucosa **(D–F)** at proximal jejunum, ileum, and colon, respectively, of calves supplemented (SCB) or not (CON) from birth until 1 week of age with *Saccharomyces cerevisiae boulardii* CNCM I-1079 at 10 × 10^9^ cfu/d. Analysis of similarity (*R* = ANOSIM) were used to calculate *p*-values and to test differences in β-diversity among treatment groups.

Supplementation of newborn calves with SCB increased species richness at 1 week of age in the ileum digesta (*p* = 0.01; Figure 1A) and tended to increase species richness in colon digesta (*p* = 0.08; Figure 1A), as denoted by the Chao1 index. No differences in evenness (Shannon index) of the bacterial microbiota of calves were observed between treatments in any of the locations in the digesta samples (*p* > 0.23; Figure 1A). Additionally, SCB supplementation increased the phylogenetic diversity (PD) in the ileum digesta (*p* < 0.01; Figure 1A), as denoted by the PD faith index. In the tissue samples, no difference between groups were observed in species richness (*p* > 0.34; Figure 1B), evenness (*p* > 0.21; Figure 1B), or PD (*p* > 0.17; Figure 1B). To compare bacterial communities between groups, we used unweighted UniFrac distance matrixes in both digesta and tissue samples of the different segments of the intestine and were visualized by NMDS (Figure 2). These results revealed that the bacterial profiles generated from ileum digesta-associated communities tended to separate into distinct clusters according to treatment (ANOSIM *R* = 0.20, *p* = 0.02; Figure 2B), with no differences observed in the other regions of the intestine both for mucosa and digesta.

### 3.2. *Saccharomyces cerevisiae* concentrations, LEfSe, and relative abundance of discriminant taxa

Copy numbers of 26S rRNA *S. cerevisiae* gene were detected in the digesta of all intestinal compartments measured. Supplementation of SCB in milk significantly increased the density of *S. cerevisiae* per g of digesta in jejunum, ileum, and colon compared with non-supplemented calves (*p* < 0.001; Table 1).

**Table 1:** Concentration of *Saccharomyces cerevisiae* in the gut digesta of 1-wk-old calves in response to the supplementation of *Saccharomyces cerevisiae boulardii* CNCM I-1079.

	Treatment^2^			*p-*value^3^		
Concentration in digesta, Log^10^ copy /g of sample	CON	SCB	SEM	Location	TRT	TRT*Location
*Saccharomyces cerevisiae* ^1^				<0.001	<0.001	<0.001
Proximal-Jejunum	4.97	5.75	0.16		<0.001	
Ileum	5.03	5.93	0.23		<0.001	
Colon	3.58	5.85	0.26		<0.001	

^1^Copy number of 26S rRNA *Saccharomyces cerevisiae* gene per gram of digesta.

^2^CON = nonsupplemented calves; SCB = calves supplemented with *Saccharomyces cerevisiae boulardii* CNCM I-1079 at 10 × 10^9^ cfu/d from birth until 1 week of age. Values are least square means. ^3^TRT = Treatment.

**Figure 3:**
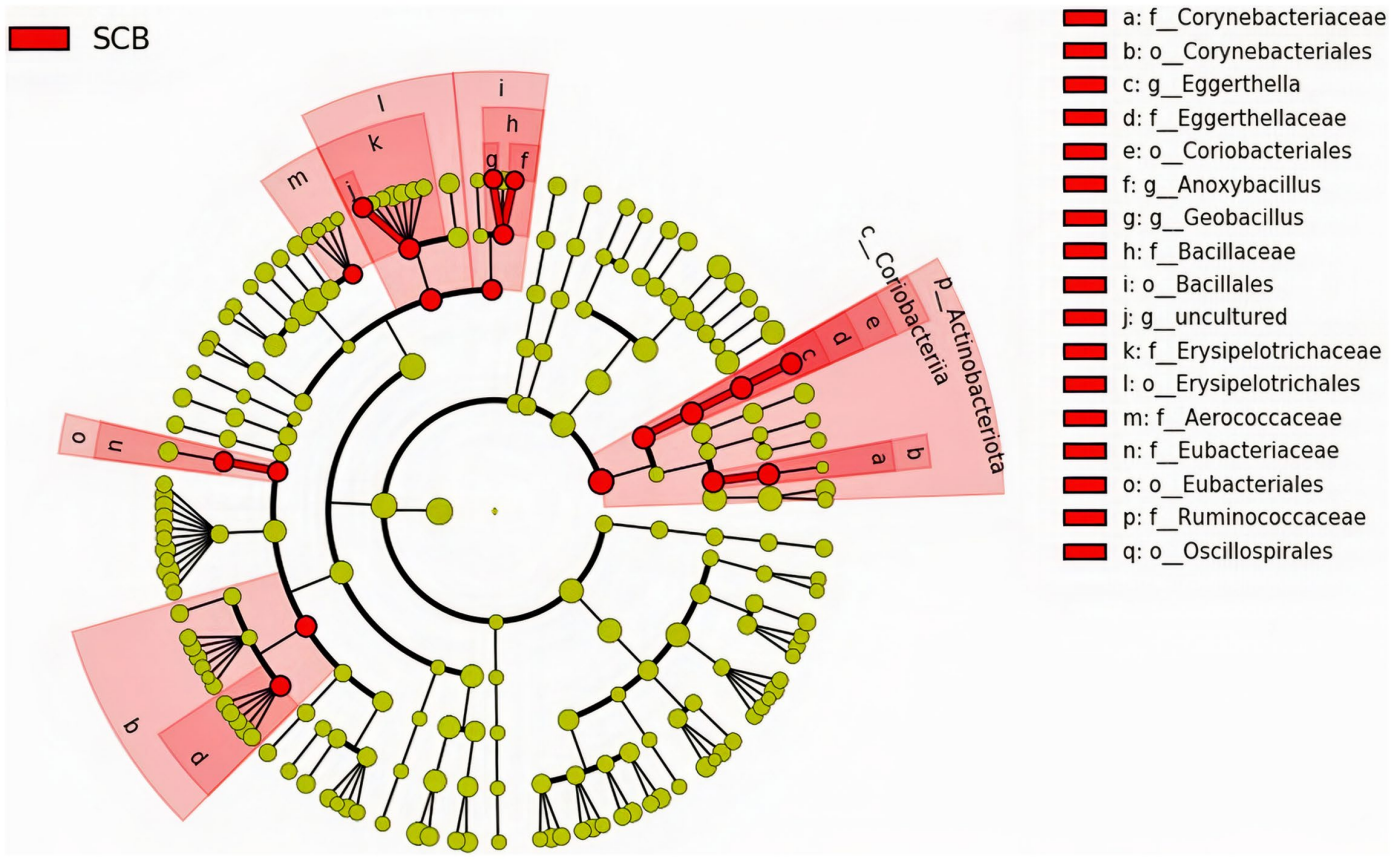
The linear discriminant analysis effect size (LEfSe) circular cladogram generated from the ileum digesta of ileum bacterial communities from phylum to genus level identified as discriminating taxa for calves supplemented with *Saccharomyces cerevisiae boulardii* CNCM I-1079 at 10 × 10^9^ cfu/d from birth to 1 week of age (SCB), and non-supplemented calves (CON).

**Figure 4:**
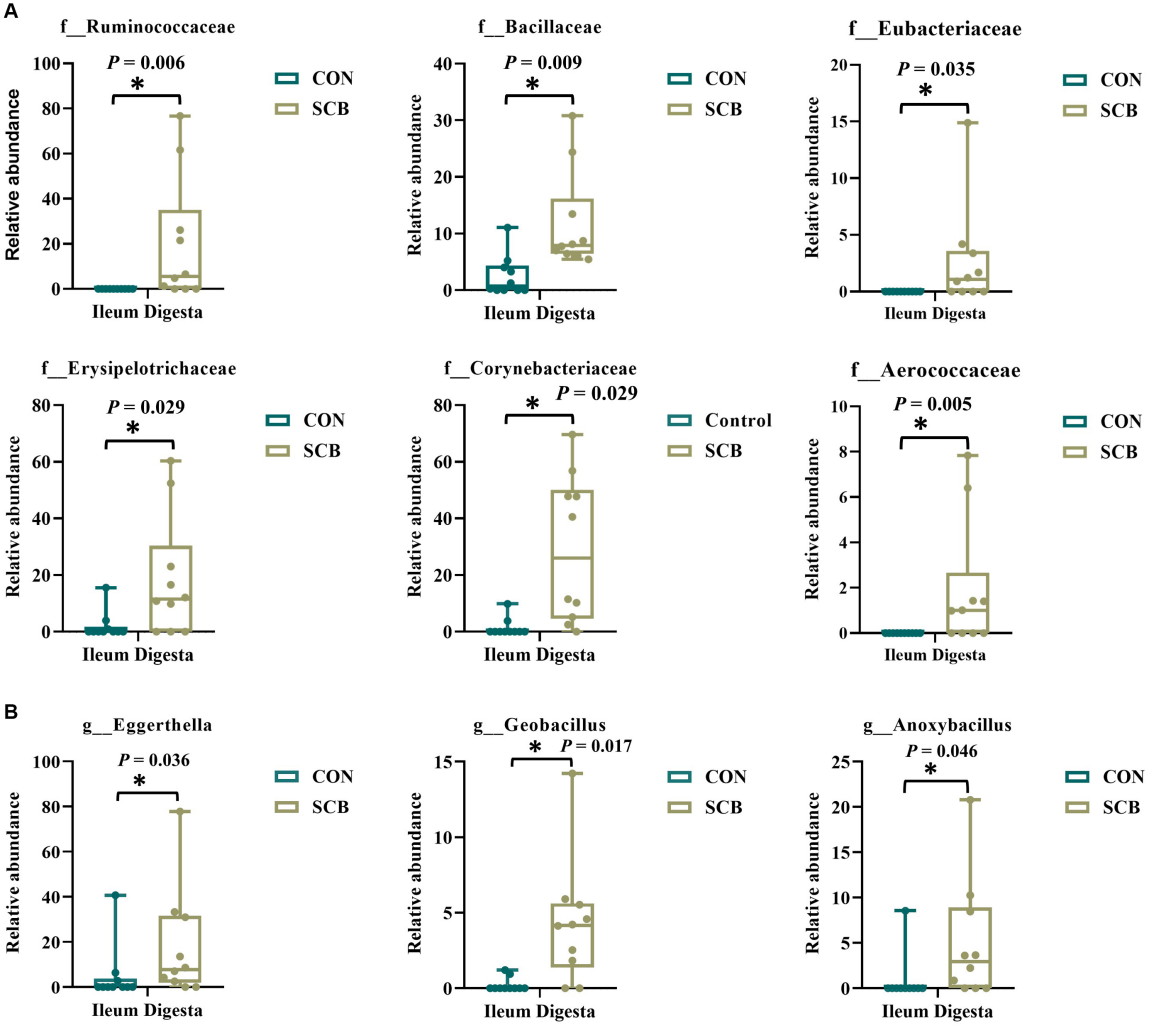
Relative abundance of discriminating taxa based on the LEfSe analysis at the family level **(A)**, and genus level **(B)** extracted from the ASV feature table compiled using the SILVA database from calves supplemented with *Saccharomyces cerevisiae boulardii* CNCM I-1079 at 10 × 10^9^ cfu/d from birth to 1 week of age (SCB), and non-supplemented calves (CON). Relative abundance is presented as box and dot plots. The lines, boxes, and whiskers in the box plots represent the median, and 25th, and 75th percentiles, and the min-to-max distribution of replicate values, respectively. Data were analyzed using the Mann–Whitney *U* test. * Indicates a significant difference between CON and SCB groups (*p* < 0.05).

We performed a linear discriminant analysis coupled with effect size measurements (LEfSe) in ileal digesta samples given that it was the only location and sample type where differences in species richness, phylogenetic diversity, and clustering of the bacterial community according to treatment were observed (Figure 1A). Results from LEfSe analysis were used to generate a circular cladogram for the ileal digesta microbiota and to identify the differentially abundant taxa between treatments (Figure 3). The LEfSe analysis in ileum digesta samples indicated that the phyla *Actinobacteriota*, including the families *Corynebacteriaceae*, *Eggerthellaceae*, as well as the families *Bacillaceae*, *Erysipelotrichaceae*, *Aerococcaceae*, *Eubacteriaceae*, and *Ruminococcaceae* were among the discriminatory taxa between CON and SCB groups. Next, the relative abundances of the discriminatory taxa between treatments were compared. The relative abundance of the families *Ruminococcaceae*, *Bacillaceae*, *Eubacteriaceae*, *Corynebacteriaceae*, *Eggerthellaceae*, *Erysipelotrichaceae*, *Aerococcaceae*, and *Eubacteriaceae* (*p* < 0.04; Figure 4A), as well as members of the genus E*ggerthella*, *Geobacillus*, and *Anoxybacillus* were significantly increased in the SCB group (*p* < 0.05; Figure 4B) compared with CON.

### 3.3. Network analysis

**Figure 5:**
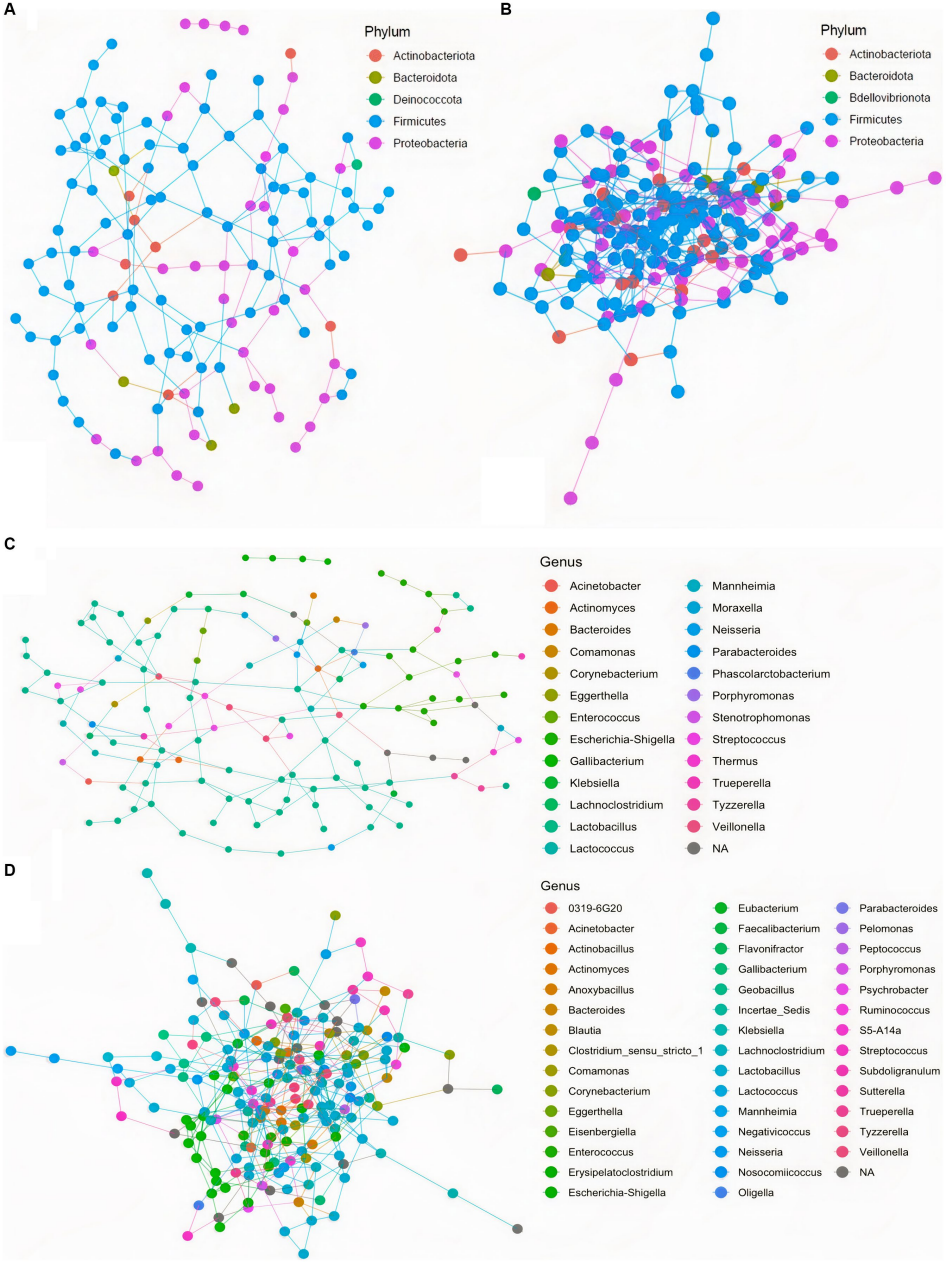
Co-occurrence network analysis using SPIEC-EASI used to infer the influence of *Saccharomyces cerevisiae boulardii* CNCM I-1079 supplementation on the microbial ecological networks in ileum digesta, at the phylum, and genus level. CON = non-supplemented calves at phylum level **(A)**, and at genus level **(C)**; SCB = calves supplemented with *Saccharomyces cerevisiae boulardii* CNCM I-1079 at 10 × 10^9^ cfu/d from birth to 1 week of age at phylum level **(B)**, and at genus level **(D)**.

**Figure 6:**
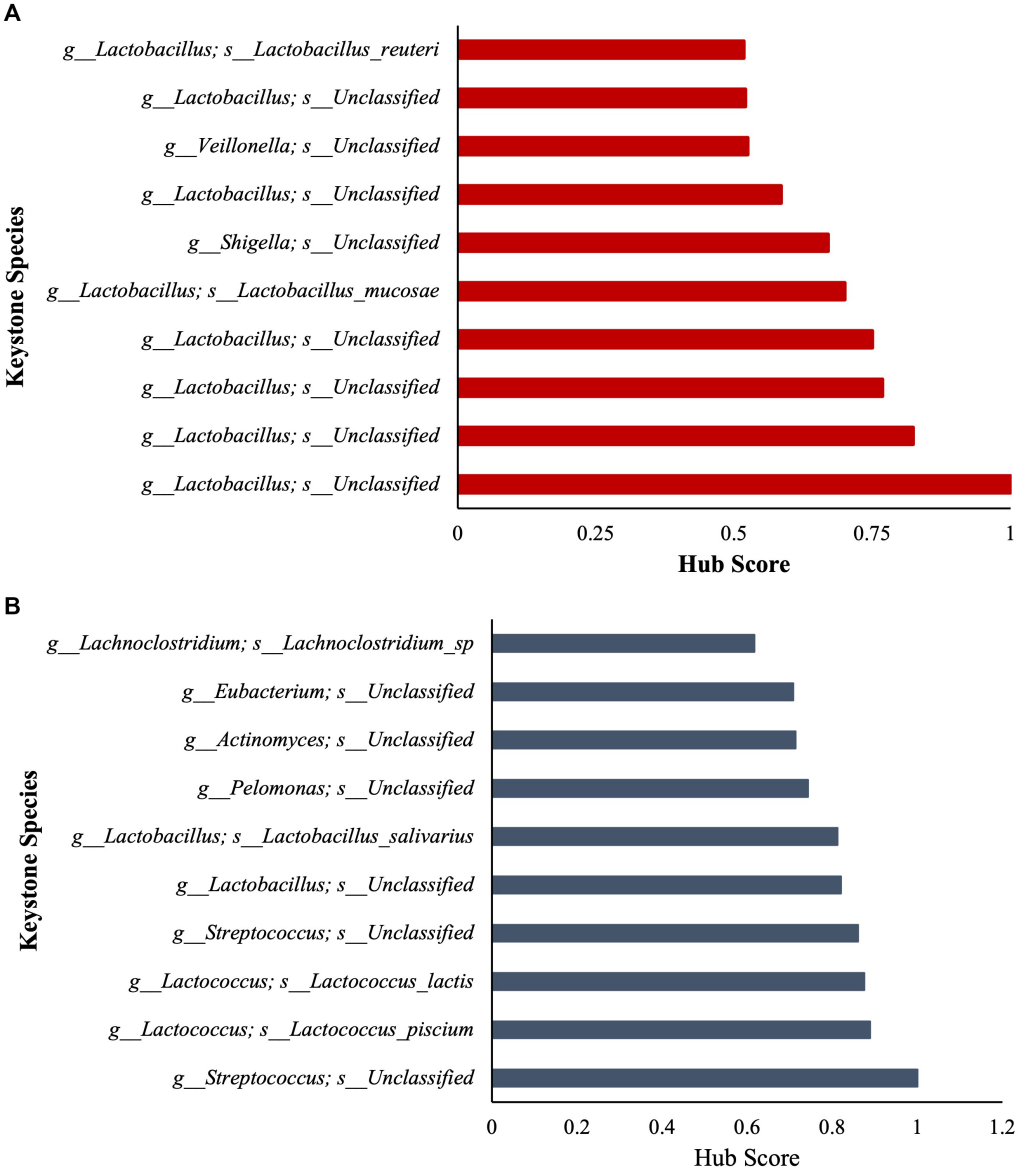
Stacked bar charts show the relative contribution of the 10 most important keystone species based on Hub score in ileum digesta within the microbiota of each treatment group (Higher hub score denotes an increased level of interconnectivity within the network). CON = non-supplemented calves **(A)**; SCB = calves supplemented with *Saccharomyces cerevisiae boulardii* CNCM I-1079 at 10 × 10^9^ cfu/d **(B)** from birth to 1 week of age.

To further assess community ecological parameters, we performed a co-occurrence network analysis using SPIEC-EASI to infer microbial ecological networks differences between the CON and SCB groups in ileal digesta. The resulting network revealed that probiotic supplementation increased the microbial interconnectivity between nodes in the network as denoted by the increase in total edges within the SCB network (417 edges) compared to CON (176 edges), and the number of total nodes (SCB = 198 vs. CON = 135) present in the network (Figure 5). In addition, the keystone species (composed of the 10 taxa most central to the microbiota networks according to the hub scores) were different between groups and show that SCB increased the number of different taxa at the genus level that are central to the microbiota network (Figure 6). For instance, the keystone species in CON group consisted primarily of bacteria from the genus *Lactobacillus*, *Escherichia Shigella*, and *Veillonella* (Figure 6A, Hub Score > 0.6), whereas the SCB group consisted of *Streptococcus*, *Lactococcus*, *Lactobacillus*, *Pelomonas*, *Actinomyces*, *Eubacterium*, *Lachnoclostridium* (Figure 6B, Hub Score > 0.6). Furthermore, the average hub scores were higher for those keystone species in SCB compared with CON groups (0.75 vs. 0.60), suggesting that these keystone species were not only different between treatment groups, but were also more interconnected within their ecological networks.

### 3.4. Secretory IgA concentrations, and short-chain fatty acid profile

**Figure 7:**
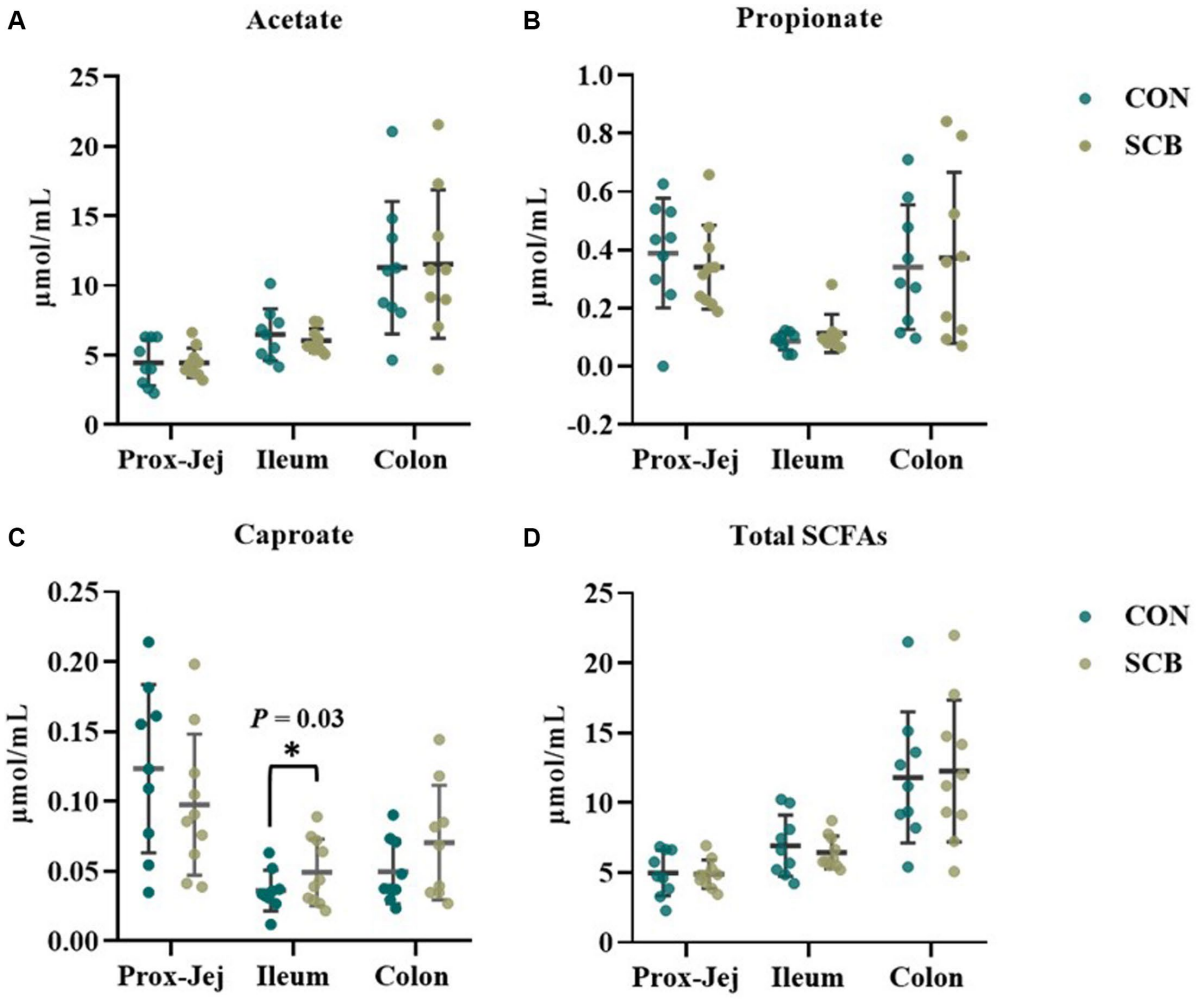
Short chain fatty acid (SCFA) profile in digesta from proximal jejunum (Prox-Jej), ileum, and colon in response to the supplementation of *Saccharomyces cerevisiae boulardii* CNCM I-1079 at 10 × 10^9^ cfu/d (SCB) from birth until 1 week of age or not supplemented calves (CON). Values are means ± SD. * Indicates a significant difference between CON and SCB groups (*p* < 0.05).

The concentrations of sIgA was measured in the digesta of distal jejunum, Ileum, Colon, and on feces. Supplementation of SCB significantly increased sIgA concentrations in the ileum, and colon digesta (*p* < 0.001; Table 2). Concentrations of total SCFAs, acetate, propionate, and caproic acid in proximal jejunum, ileum, and colon were not affected by treatment (*p* ≥ 0.25; Figure 7; Supplementary Table S3), nor the acetate to propionate ratio (*p* = 0.80; Supplementary Table 3). However, the acetate concentration was higher in the colon (*p* < 0.01; Figure 7A; Supplementary Table S3) compared to the ileum and proximal jejunum. Furthermore, when doing pairwise comparison of treatment effects by gut location and adjusted by Tukey for multiple comparisons, the concentration of caproic acid was higher in the ileum for SCB compared to CON (*p* < 0.03; Figure 7C; Supplementary Table S3).

**Table 2:** Secretory IgA (sIgA) concentration in the different segments of the gut of 1-wk-old calves (*n* = 20) in response to supplementation of *Saccharomyces cerevisiae boulardii* CNCM I-1079.

	Treatments^1^			*p*-value^2^		
	CON	SCB	SEM	Location	TRT	TRT*Location
IgA, mg/g DM				<0.001	<0.001	<0.001
Distal-Jejunum	0.2	0.22	0.12			0.938
Ileum	1.18	1.98	0.12			<0.001
Colon	0.59	1.45	0.12			<0.001
Feces	0.64	0.71	0.14			0.707

^1^CON = nonsupplemented calves; SCB = calves supplemented with *Saccharomyces cerevisiae boulardii* CNCM I-1079 at 10 × 10^9^ cfu/d from birth until 1 week of age. Values are least square means. ^2^TRT = Treatment.

### 3.5. Correlation between physiological variables and intestinal digesta microbiota

**Figure 8:**
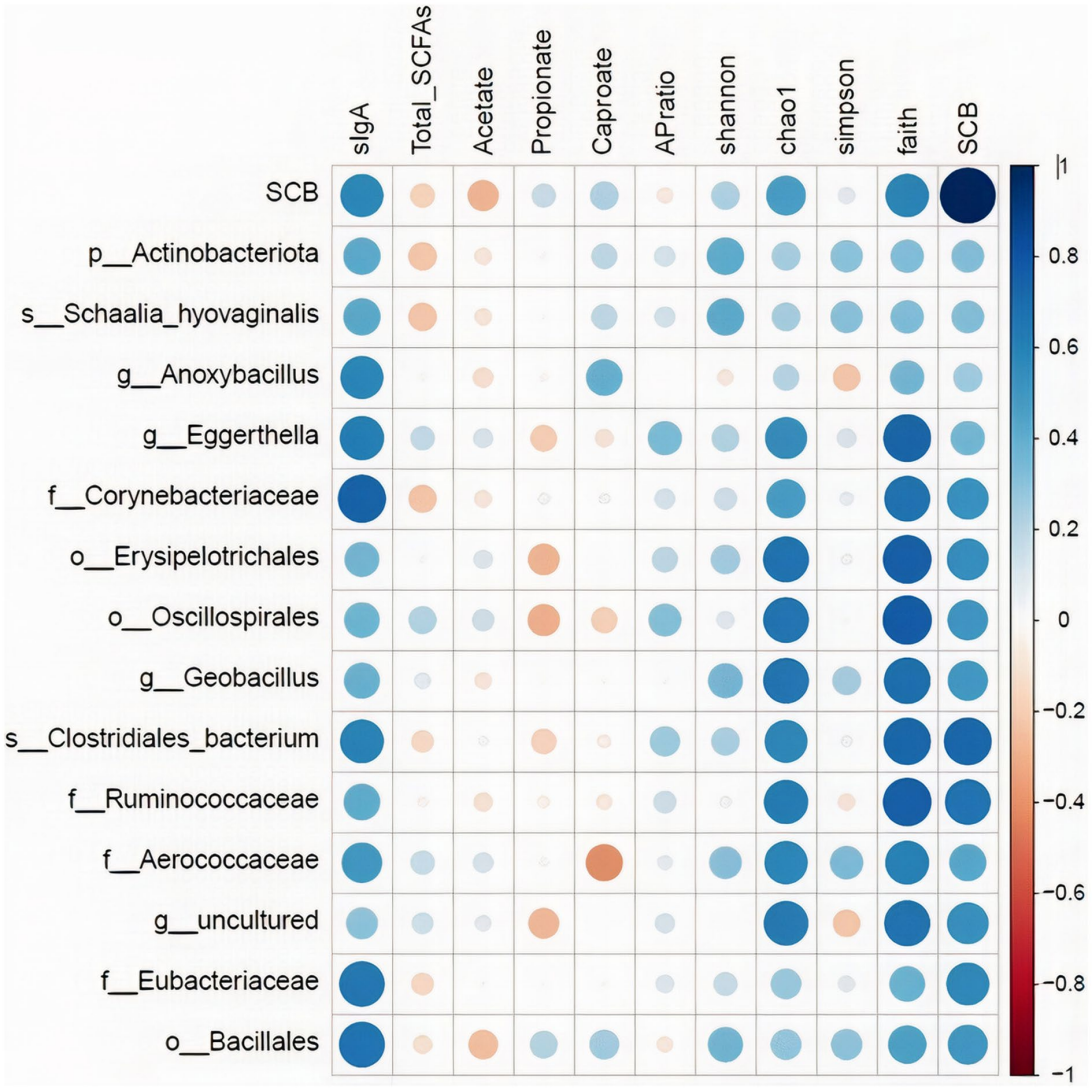
Spearman rank correlations of the relative abundance of the discriminatory taxa obtained in LEfSe in addition to the *Saccharomyces cerevisiae* gene copy numbers (SCB) in ileum digesta with: secretory IgA concentrations in ileum (sIgA-ileum mg/g DM); concentration of total short chain fatty acids (SCFAs), acetate (μmol/mL), propionate (μmol/mL), and caproate (μmol/mL) measured in ileum; and alpha diversity measures: Chao1, Shannon, Faith phylogenetic diversity (PD). Data is graphically presented in a dot plot heatmap. Strong correlations indicated by large circles and weaker correlations indicated by smaller circles. The scale colors denote whether the correlation is positive (closer to 1, blue circles) or negative (closer to −1, red circles).

The relative abundance of the discriminatory taxa obtained in LEfSe in addition to the *Saccharomyces cerevisiae* gene copy numbers in ileum digesta was then correlated to sIgA, as well as short chain fatty acids profile, and alpha diversity indices in the same segment and sample type using spearman rank correlations (Figure 8). The gene copy numbers of SCB were positively correlated with the concentration of sIgA in ileum digesta (*p* < 0.01; Spearman’ *ρ* = 0.58). In addition, the relative abundance of the family *Corynebacteriaceae* (*p* < 0.01; Spearman’ *ρ* = 0.73), and *Eubacteriaceae* (*p* < 0.01; Spearman’ *ρ* = 0.65) were positively correlated with the concentrations of sIgA in the ileum digesta. Additionally, the relative abundance of bacterial families *Ruminococcaceae* (*p* < 0.01; Spearman’ *ρ* = 0.62), *Aerococcaceae* (*p* < 0.01; Spearman’ *ρ* = 0.59), and *Corynebacteriaceae* (*p* = 0.03; Spearman’ *ρ* = 0.48), and genus *Eggerthella* (*p* = 0.01; Spearman’ *ρ* = 0.55) were positively correlated with species richness (Chao1 index). Total SCFAs, acetate, propionate, and their ratio were not correlated with SCB gene copy numbers nor with any of the differentially abundant taxa between groups (Figure 8).

## 4. Discussion

Our study revealed that supplementing calves with the strain of *Saccharomyces cerevisiae boulardii* CNCM I-1079 from birth to 1 week of age, increased species richness and phylogenetic diversity in addition to expediting the transition to a more interconnected bacterial community in the ileum, two key features of later stages of primary ecological succession (Connell and Slatyer, 1977). Because the major differences in alpha and beta diversity were observed in the ileum digesta, which also coincides with the intestinal region where the differences in sIgA production were observed in our companion paper (Villot et al., 2020), we decided to focus the rest of our exploratory analysis on this region of the intestine. We performed a LEfSe analysis to determine which discriminatory bacterial taxa were different between the treatments. This analysis revealed that there were several differentially abundant taxa between treatments and that SCB increased the relative abundance of members of the phylum *Actinobacteria*, family *Eubacteriaceae*, *Corynebacteriaceae*, *Eggerthellaceae*, *Bacillaceae*, and *Ruminococcaceae*. Members of the *Actinobacteria* phylum have been previously reported to increase in cecum and colon of piglets supplemented with SCB (Brousseau et al., 2015; Kiros et al., 2019). In addition, bacterial members of this group are major utilizers of lactose in the neonatal human gut and can modulate the pathogenicity of some bacterial groups (Bag et al., 2017). In addition, members of the *Eubacteriaceae* and *Ruminococcaceae* family are important producers of SCFAs and have been shown to be increased by supplementation with the live yeast *Saccharomyces cerevisiae* to neonatal piglets (Kiros et al., 2019), and with SCB to weaned piglets (Zhang et al., 2020). Furthermore, members of the *Ruminococcaceae* family can utilize yeast cell wall components, such as mannose, chitin, 1,3-β-glucan and 1,6-β-glucan (Czerucka et al., 2007), as fermentation substrates to produce SCFAs. As key bacterial metabolites produced by gut bacteria, SCFAs play a crucial role in linking microbiota composition and various biological effects at the mucosal level. In our study, although we observed changes in the relative abundance of major SCFAs producing bacteria, we did not observe variation in the production of SCFAs, other than a small increase in caproate. As expected, the highest concentrations of SCFA were observed in the colon, where most of the fermentation occurs in the intestine. However, the diet of the calves was composed strictly of milk replacer, that offers virtually no fiber to stimulate production of SCFAs in the lower gut, and even though SCFA producing bacteria were present in the intestine and lower gut, there was very minimal substrate available to ferment, as reflected in the low concentrations of SCFAs found in this study. It has been previously reported that in the rumen of neonatal calves of less than 1 week of age, functionally and metabolically active microbiota with the ability to ferment carbohydrates even before exposure to solid diet are present and are suggested to be vertically transferred by the dam (Malmuthuge et al., 2015). The small and large intestine of a newborn could also harbor functionally active SCFA producing bacteria even before exposure to solid diet.

We then identified keystone bacterial species that were central to the structure of the microbial community in the ileum digesta by using a co-occurrence network analysis, in addition to a multivariate associative test. In community ecology, keystone species are a group of species that play a crucial role in community organization, function, and diversity, and its loss results in the deterioration of the ecosystem function (Paine, 1966; Amit and Bashan, 2023). In our study, we observed that the keystone species were different between treatment groups and that SCB increased the number of different taxa that are central to the microbiota network. In addition, the level of interconnectivity within the network was higher in the SCB group compared to CON, suggesting a more stable microbial network, an important ecological feature that provides resistance to invasion by opportunistic pathogenic bacteria (Masi and Stewart, 2019; Samara et al., 2022). Furthermore, SCB keystone species were composed of members from the *Lactobacillus*, *Streptococcus*, *Lactococcus*, *Eubacterium*, *Lachnoclostridium*, *Actinomyces*, and *Pelomonas*, whereas non-supplemented calves’ keystone species were only composed of members of the *Veillonella*, *Lactobacillus* and *Shigella* genus. Members of the *Shigella* genus contains pathogenic-facultative anaerobe bacteria that can promote intestinal tissue damage, and gastrointestinal disorders, and its relative abundance has been shown to be higher in diarrheic calves (Villot et al., 2019). Supplementation of SCB has been shown to reduce the pathogenicity of *Shigella* infection, but this protective effect seems to be mediated by a reduction in intestinal lesions and bacterial infiltration rather than by a direct reduction in this bacterial level in the intestinal population (Mumy et al., 2008). In this study, SCB appears to have modulated the establishment of keystone species in the neonatal calf’s gut and increase the ecosystem resistance against *Shigella* infection. It has been previously reported that *Saccharomyces cerevisiae* reduces oxygen levels and redox potential in the rumen of lambs (Chaucheyras-Durand and Fonty, 2002) and adult ruminants (Marden et al., 2008), thereby improving the conditions for strict anaerobes to thrive. In this study SCB could have reduced oxygen levels in the intestine and promote the establishment of strict anaerobic bacteria faster than in the non-supplemented group, preventing facultative anaerobic pathogens to grow. These results provide an explanation as to why SCB supplementation seems to function best during scenarios of high disease pressure (Villot et al., 2019) by creating a more stable bacterial community, therefore, preventing invasion with opportunistic pathogenic bacteria.

We observed that the probiotic-driven increased diversity of the intestinal bacterial microbiota was highly correlated with the enhanced secretory IgA capacity of the ileum. Furthermore, the relative abundance of the family *Corynebacteriaceae* and *Eubacteriaceae*, were among the discriminatory groups between treatments and an increase in relative abundance of these taxa in the SCB group was positively correlated with the concentration of sIgA in the ileum. This data suggests that the greater microbial diversity and the ecological attributes of a more diverse and interconnected microbiota promoted by SCB might have provided the necessary signals for stimulating the increased production of sIgA in the ileum. In ruminants, the ileum possesses continuous Peyer’s patches that serve as an immune induction site for the intestinal mucosa (Snoeck et al., 2006; Fries et al., 2011; Villot et al., 2020). Activation and class switching of B cells occur in organized lymphoid structures, such as the Peyer patches, and its development and proper function depends on signals provided by the intestinal microbiota (Stavnezer et al., 2008; Vergani et al., 2022). Secretory IgA binds luminal bacteria and promotes its clearance from the gut, preventing direct contact of bacteria with the epithelial cell surface, to protect the gut mucosal surface from unnecessary immune activation and inflammation, in a process known as immune exclusion (Corthésy et al., 2006; Pabst et al., 2016). In addition, sIgA enhances transport of antigens and microorganisms from the lumen across the mucosal barrier through M cells into peyer’s patches and mesenteric lymph nodes, which in turn, improves antigen presentation to T cells, thus promoting immune induction to specific non-invasive microorganisms (Mantis et al., 2002). Therefore, sIgA helps to modulate the composition of the intestinal microbiota, as sIgA deficient mice pups develop a different intestinal microbiota from that of normal non-deficient pups, and that difference persists into adulthood (Fransen et al., 2015). Supplementation of SCB to neonates has been consistently shown to promote sIgA production in the intestine increasing the host’s resistance to enteropathogenic bacterial infections (Rodrigues et al., 2000; Qamar et al., 2001), resulting in a reduction in the severity of diarrhea in calves (Villot et al., 2019). We speculate that the increase in microbial diversity stimulated by SCB supplementation in the ileum could have provided the necessary signals to stimulate sIgA production and secretion, and that the increase in sIgA could have partially mediated the changes in the composition of the ileal microbiota of the supplemented calves.

This study provides further evidence that supplementing newborn calves with SCB can promote the establishment of a healthy intestinal microbiota. Supplementation of SCB to newborn calves from birth until 1 week of life increased both the species richness and phylogenetic diversity of the infant bacterial microbiota in the ileum, resulting in a more mature and stable bacterial microbiota when compared to the non supplemented group. In addition, SCB modulated the establishment of keystone species in the neonatal calf’s gut, possibly increasing resistance against opportunistic pathogenic bacteria, like *Escherichia Shigella*. The positive correlation between the increase in microbiota diversity and the increase in sIgA of the ileum mucosa provides further evidence that the calf’s IgA secretory system relies on the development of a stable and diverse microbial community to provide the necessary signals to enhance IgA production and secretion. Future studies should focus on the long-term effects on the health and performance of these early life interventions.

## Data availability statement

The data presented in the study are deposited in the NCBI SRA repository, accession number PRJNA1013102.

## Ethics statement

The animal study was approved by Canadian Council of Animal Care. The study was conducted in accordance with the local legislation and institutional requirements.

## Author contributions

CV and MS conceived and designed the experiment. LR analyzed the data and wrote the manuscript. NM, LG, RG, and RA-H assisted with data analysis, data interpretation, and manuscript review. All authors contributed to the article and approved the submitted version.

## Conflict of interest

The authors declare that the research was conducted in the absence of any commercial or financial relationships that could be construed as a potential conflict of interest.

## Publisher’s note

All claims expressed in this article are solely those of the authors and do not necessarily represent those of their affiliated organizations, or those of the publisher, the editors and the reviewers. Any product that may be evaluated in this article, or claim that may be made by its manufacturer, is not guaranteed or endorsed by the publisher.
