# Supplementary material for: Saccharomyces cerevisiae boulardii accelerates intestinal microbiota maturation and is correlated with increased secretory IgA production in neonatal dairy calves
Source: Front Microbiol. 2023 Sep 19;14:1129250. doi: 10.3389/fmicb.2023.1129250 (PMC10546063; doi:10.3389/fmicb.2023.1129250)
Supplement: Supplementary file 1 [file Table_1.DOCX]

Supplementary Material

Saccharomyces cerevisiae boulardii accelerates intestinal microbiota maturation and is correlated with increased secretory IgA production in neonatal dairy calves

L.R. Cangiano*, C. Villot, R. Amorin-Hegedus, N. Malmuthuge, R.J. Gruninger, L. L. Guan, and M. A. Steele

*** Correspondence:** Corresponding Author: masteele@uoguelph.ca

# Supplementary Data

| **Table 1:** Intestinal digesta alpha diversity metrics | | | | |
| --- | --- | --- | --- | --- |
|  | Treatments^1^ | |  |  |
|  | CON | SCB | SEM^2^ | *P*-Value |
| **Proximal Jejunum** |  |  |  |  |
| Shannon | 3.93 | 3.83 | 1.02 | 0.55 |
| Chao1 | 92 | 92.18 | 31.76 | 0.82 |
| Simpson | 0.8 | 0.79 | 0.17 | 0.60 |
| Faith | 14.19 | 13.2 | 2.50 | 0.45 |
| **Ileum** |  |  |  |  |
| Shannon | 3.63 | 4.07 | 0.66 | 0.23 |
| Chao1 | 75.2 | 118.23 | 33.02 | 0.01 |
| Simpson | 0.82 | 0.85 | 0.09 | 0.60 |
| Faith | 11.2 | 14.57 | 2.54 | < 0.01 |
| **Colon** |  |  |  |  |
| Shannon | 3.33 | 3.68 | 0.61 | 0.36 |
| Chao1 | 89.04 | 104.84 | 17.60 | 0.08 |
| Simpson | 0.77 | 0.8 | 0.10 | 0.65 |
| Faith | 10.13 | 11.1 | 1.36 | 0.15 |

^1^CON = no supplemented calves; SCB = calves supplemented with Saccharomyces cerevisiae boulardii CNCM I-1079 from birth until 1 week of age. ^2^Standard error of the mean. ^3^Significance declared when P ≤ 0.05.

| **Table 2:** Intestinal tissue alpha diversity metrics | | | | |
| --- | --- | --- | --- | --- |
|  | Treatments^1^ | |  |  |
|  | CON | SCB | SEM^2^ | *P*-Value |
| **Proximal Jejunum** |  |  |  |  |
| Shannon | 3.93 | 2.91 | 1.12 | 0.21 |
| Chao1 | 34.5 | 20.4 | 19.75 | 0.09 |
| Simpson | 0.9 | 0.75 | 0.13 | 0.16 |
| Faith | 5.33 | 3.7 | 2.17 | 0.25 |
| **Ileum** |  |  |  |  |
| Shannon | 3.56 | 3.86 | 0.77 | 0.22 |
| Chao1 | 28.1 | 42.5 | 24.03 | 0.34 |
| Simpson | 0.86 | 0.87 | 0.067 | 0.76 |
| Faith | 3.66 | 5.02 | 1.81 | 0.17 |
| **Colon** |  |  |  |  |
| Shannon | 3.05 | 2.82 | 1.11 | 0.74 |
| Chao1 | 30.31 | 36 | 19.48 | 0.52 |
| Simpson | 0.75 | 0.68 | 0.22 | 0.94 |
| Faith | 3.26 | 4.02 | 1.50 | 0.33 |

^1^CON = no supplemented calves; SCB = calves supplemented with Saccharomyces cerevisiae boulardii CNCM I-1079 from birth until 1 week of age. ^2^Standard error of the mean. ^3^Significance declared when P ≤ 0.05.

Table 3: Short chain fatty acid (SCFA) profile of intestinal digesta from control and calves supplemented with SCB

|  | Treatment^1^ | | |  | |  | |  | | *P*-Value | | | | | | | |  |
| --- | --- | --- | --- | --- | --- | --- | --- | --- | --- | --- | --- | --- | --- | --- | --- | --- | --- | --- |
| Measurement | CON | SCB | |  | | SEM^2^ | |  | | Treatment | | Location | | Treatment × Location | | CON vs. SCB^3^ | |  |
| Acetic acid, μmol/mL |  |  | |  | |  | |  | | 0.988 | | < 0.01 | | 0.933 | |  | |  |
| Proximal Jejunum | 4.218 | | 4.438 | |  | | 1.044 | |  | |  | |  | |  | | 0.883 | |
| Ileum | 6.460 | | 6.023 | |  | | 1.108 | |  | |  | |  | |  | | 0.768 | |
| Colon | 11.276 | | 11.533 | |  | | 1.044 | |  | |  | |  | |  | | 0.863 | |
| Propionic acid, μmol/mL |  | |  | |  | |  | |  | | 0.397 | | 0.627 | | 0.502 | |  | |
| Proximal Jejunum | 0.382 | | 0.341 | |  | | 0.133 | |  | |  | |  | |  | | 0.865 | |
| Ileum | 0.382 | | 0.113 | |  | | 0.141 | |  | |  | |  | |  | | 0.827 | |
| Colon | 0.341 | | 0.373 | |  | | 0.133 | |  | |  | |  | |  | | 0.158 | |
| Ace/Prop^4^ |  | |  | |  | |  | |  | | 0.809 | | < 0.01 | | 0.835 | |  | |
| Proximal Jejunum | 10.864 | | 15.1103 | |  | | 10.248 | |  | |  | |  | |  | | 0.641 | |
| Ileum | 66.963 | | 62.0235 | |  | | 10.869 | |  | |  | |  | |  | | 0.772 | |
| Colon | 50.844 | | 57.6525 | |  | | 10.248 | |  | |  | |  | |  | | 0.735 | |
| Caproic acid, μmol/mL |  | |  | |  | |  | |  | | 0.250 | | 0.077 | | 0.175 | |  | |
| Proximal Jejunum | 0.407 | | 0.098 | |  | | 0.098 | |  | |  | |  | |  | | 0.882 | |
| Ileum | 0.036 | | 0.045 | |  | | 0.104 | |  | |  | |  | |  | | 0.031 | |
| Colon | 0.061 | | 0.082 | |  | | 0.098 | |  | |  | |  | |  | | 0.950 | |
| Total SCFAs, μmol/mL |  | |  | |  | |  | |  | | 0.419 | | < 0.01 | | 0.707 | |  | |
| Proximal Jejunum | 6.691 | | 4.877 | |  | | 1.190 | |  | |  | |  | |  | | 0.916 | |
| Ileum | 6.926 | | 6.181 | |  | | 1.263 | |  | |  | |  | |  | | 0.289 | |
| Colon | 11.811 | | 11.990 | |  | | 1.190 | |  | |  | |  | |  | | 0.660 | |

^1^CON = no supplemented calves; SCB = calves supplemented with Saccharomyces cerevisiae boulardii CNCM I-1079 from birth until 1 week of age. ^2^Standard error of the mean. ^3^Significance declared when P ≤ 0.05. ^4^Acetate to propionate ratio.
